# Evaluation of the quality of information provided by ChatGPT on distal biceps repair surgery

**DOI:** 10.1007/s11845-026-04452-6

**Published:** 2026-06-05

**Authors:** Conor J. Kilkenny, Sean P. Whealan, Danilo Vukanic, Alex Stapleton, Kieran O’Sullivan, Eoghan T. Hurly, Diarmuid C. Molony

**Affiliations:** RCSI: RCSI Dublin, Dublin, Ireland

**Keywords:** Artificial intelligence, ChatGPT, Distal biceps repair, Distal biceps tendon rupture, Patient education, Readability

## Abstract

**Background:**

Distal biceps tendon rupture is a rare injury, typically occurring during forceful, eccentric contraction of the biceps brachii muscle. This study aimed to assess the information AI software (ChatGPT) provides when searching for distal bicep injuries and their management. Using standardised scoring systems, we evaluated the quality of the information provided, its trustworthiness, and its readability.

**Methods:**

An open AI model (ChatGPT) was used to answer 25 commonly asked questions from patients about distal biceps surgery. These answers were evaluated for medical accuracy, quality, and readability using the JAMA Benchmark criteria, DISCERN score, Flesch-Kincaid Reading Ease Score (FRES), and Grade Level (FKGL).

**Results:**

The JAMA Benchmark criteria score was 0, the lowest score, indicating no reliable resources cited. The DISCERN score was 44.3, which is considered a good score. The areas in the open AI model that did not achieve full marks were related to the lack of available source material used to compile the answers and, finally, some shortcomings with information not fully supported by the literature. The FRES was 38.5, and the FKGL was at a college reading level.

**Conclusion:**

A high reading level was required to comprehend the information provided by ChatGPT about distal biceps repair, and the evidence supplied was of fair quality. With no citations provided, it remains unclear where these answers originate. However, ChatGPT continued to safety-net patients by encouraging the importance of further discussion with a surgeon. More high-quality sources that patients can easily comprehend are required to educate patients concerning distal bicep repair.

## Introduction

Distal biceps tendon rupture is a relatively rare but functionally important injury, typically occurring when a strong eccentric load is applied to the biceps brachii. The resulting deficit in elbow flexion strength and forearm supination can be substantial [1]. The condition predominantly affects middle-aged men, with an estimated incidence of 1.2–9.6 per 100,000 person-years [1]. For patients with high functional demands, operative repair is generally recommended, as it offers better restoration of strength and endurance than non-operative management [2].

Informed consent is a cornerstone of safe surgical practice [3]. Keulers et al. showed that surgeons often underestimate how much information patients would like to receive before surgery, leading many individuals to seek additional material from other sources [4]. Increasingly, patients supplement consultation-room discussions with information they obtain online, including content generated by artificial intelligence (AI) tools such as ChatGPT. At present, there are no universal regulatory standards ensuring the accuracy or transparency of information produced by large language models, and human oversight remains necessary to detect errors or misinterpretations [5].

Literacy and health literacy vary widely in the general population. AI systems typically provide a single, non-tailored response that does not adjust to the user’s reading ability.

Prior studies examining AI-generated content in various medical and orthopaedic domains have reported that, although the information may be broadly accurate, it is frequently written at a level that is too advanced for many patients [6–8]. ChatGPT has previously been evaluated for topics such as shoulder stabilisation and other upper-limb procedures [9, 10], but distal biceps tendon rupture has not yet been specifically examined.

The aim of this study was to assess the quality, reliability and readability of ChatGPT’s responses to common patient questions about distal biceps tendon rupture and repair. We hypothesised that ChatGPT would provide information of fair overall quality, but at a reading level higher than that recommended for patient education materials.

## Materials & methods

### Artificial intelligence evaluation

On 25 May 2024, the ChatGPT interface was accessed and a set of 25 frequently asked patient questions relating to distal biceps tendon rupture and its operative management was submitted. The questions, listed in full in Appendix A, were based on previously published work exploring patient information needs after distal biceps surgery, supplemented by themes commonly encountered in clinical practice. All responses were generated in a single session using the same version of ChatGPT available on that date.

Three investigators (CJK, SPW and AS) independently reviewed each answer. The responses were evaluated for informational quality using the DISCERN instrument and JAMA benchmark criteria, and for readability using the FRES and FKGL metrics.

### Quality analysis

#### DISCERN

The DISCERN questionnaire is a validated tool used to assess the quality of written information about treatment options [11]. It comprises 16 questions, commonly grouped into three sections: reliability of the publication (8 questions), quality of information about treatment choices (7 questions), and a final overall quality rating. Following the approach of Weil et al. [12], we excluded the last, global-rating item from analysis. Total DISCERN scores were interpreted as: 63–75 (excellent), 51–62 (good), 39–50 (fair), 27–38 (poor) and 15–26 (very poor).

#### JAMA benchmark criteria

The JAMA benchmark criteria offer a simple framework for assessing transparency and accountability in health information resources [13]. They consider four domains:

• Authorship—whether the author(s) and their credentials are clearly identified.

• Attribution—whether sources and copyright information are properly cited.

• Disclosure—whether potential conflicts of interest or sponsorship are declared.

• Currency—whether the date of creation or last update is specified.

Each domain is scored as present or absent, giving a total possible score between 0 and 4.

### Readability analysis

Readability of the ChatGPT responses was assessed using the Flesch-Kincaid Reading Ease Score (FRES) and the Flesch-Kincaid Grade Level (FKGL). Scores were calculated using an online readability calculator [14]. The FRES yields values from 0 to 100, with higher scores indicating easier-to-read text and lower scores indicating more complex material. The FKGL estimates the US school grade level required to understand the text [15].

### Statistical analysis

Descriptive statistics were calculated using Microsoft Excel (Microsoft Corp., Redmond, WA, USA). Inter-rater reliability for the DISCERN scores was assessed with the kappa statistic. Agreement was interpreted according to commonly used thresholds: 0.01–0.20 (slight agreement), 0.21–0.40 (fair), 0.41–0.60 (moderate), 0.61–0.80 (substantial) and 0.81–1.00 (almost perfect) [16].

## Results

### JAMA benchmark

The overall JAMA benchmark score for the ChatGPT responses was 0. None of the answers included named authors or stated qualifications, cited references, declarations of conflicts of interest or sponsorship, or time stamps indicating when the content had been created or updated. This complete absence of these four benchmark domains highlights a lack of transparency and accountability.

### DISCERN scores

The mean DISCERN score was 44.3, which corresponds to fair quality. ChatGPT generally outlined the mechanism of injury, indications for surgery, broad treatment options and typical recovery milestones in a coherent manner. However, points were commonly lost because statements were not supported by cited evidence and some explanations were overly general. For example, operative techniques were sometimes described as “effective” without clear discussion of comparative risks and benefits.

Inter-rater reliability for DISCERN scoring was 0.75, indicating substantial agreement among the three reviewers.

### Readability and content themes

The mean FRES was 38.5, reflecting relatively difficult text, and the mean FKGL was 11.8, corresponding to a reading level consistent with late secondary school or early college education. In practical terms, this suggests that many patients would find the material challenging to interpret.

Content analysis showed that ChatGPT typically:

- Described distal biceps rupture as occurring after a sudden, forceful load on a flexed elbow.
- Recommended operative repair particularly for younger or more active patients with complete ruptures.
- Outlined a staged rehabilitation process, with a return to unrestricted activity commonly expected several months after surgery.

Nevertheless, the responses did not include references, gave limited detail about general anaesthetic risks and other peri-operative complications, and sometimes relied on non-specific statements such as suggesting that multiple operative techniques are “viable” without further elaboration. The full set of questions and unedited ChatGPT responses is presented in Appendix A.

## Discussion

The main finding of this study is that ChatGPT provided fair-quality information about distal biceps tendon rupture and repair, but at a reading level that is likely too high for a substantial proportion of patients. The responses were generally clinically reasonable and internally consistent, yet the absence of citations and authorship details limited their verifiability and overall trustworthiness.

Effective communication remains central to orthopaedic practice. Prior work has demonstrated that surgeons often underestimate the amount and complexity of information patients wish to receive before surgery [4]. At the same time, many adults have literacy levels below those required to understand complex medical text [17]. This mismatch encourages patients to seek supplementary information from external sources, particularly the internet [18, 19]. AI-driven platforms such as ChatGPT now occupy a prominent place within this information landscape.

AI tools have been proposed for diagnostic support, triage, documentation and patient education [20, 21]. However, their performance depends heavily on the quality and recency of the data on which they were trained [20–22]. As evidence evolves, previously generated responses may become outdated. In addition, large language models do not inherently distinguish between high-quality and low-quality sources unless they have been explicitly curated.

Demographic data indicate that approximately one-third of ChatGPT users are aged 25–34 years, and most are younger than 55 [23]. This overlaps closely with the typical age range in which distal biceps ruptures occur (35–54 years) [24]. Literacy surveys from Ireland and the United States show that a significant proportion of adults struggle with higher-level written material [18, 19]. Given that the FKGL in this study was 11.8, the readability of ChatGPT’s responses is likely beyond the comfort zone of many patients who might be affected by this injury.

The Health On the Net (HON) code was introduced to improve the reliability of online health information, emphasising clear authorship, justification of claims, attribution of sources and transparency about advertising [25, 26]. ChatGPT, like other large language models, does not currently fall under HON accreditation or comparable regulatory frameworks. Without such oversight, there is potential for dissemination of incomplete, biased or outdated information, with no straightforward mechanism for users to judge quality. Independent evaluation and external regulation will be necessary if AI-generated content is to be formally incorporated into patient education pathways [22].

Our findings align with previous studies examining ChatGPT in orthopaedics and sports medicine. Hurley et al. reported that ChatGPT’s responses regarding shoulder stabilisation procedures were of fair quality but written at an advanced reading level and devoid of references [9]. Yüce et al. found that ChatGPT produced moderate-to-good quality information for sports surgery topics, again with readability as a major limitation [27]. Similar concerns have been raised in other orthopaedic domains [6–8, 28].

In the specific context of distal biceps tendon repair, ChatGPT generally provided a reasonable overview of indications and expected outcomes but did not reliably emphasise the need for individualised assessment by an orthopaedic surgeon. Nuances such as patient comorbidities, occupational demands and sport participation are difficult to address in generic AI-generated text. Consequently, ChatGPT should be viewed as an adjunct source of broad information rather than a tool for personalised decision-making.

## Limitations

This study has several limitations. First, it evaluates a single AI platform at one point in time, and large language models are periodically updated without transparent version control. Future updates could alter response quality, readability or content. Second, the list of 25 questions was derived from prior literature and clinical experience, and may not encompass the full range of concerns expressed by all patients with distal biceps pathology. Third, ChatGPT cannot incorporate individual patient characteristics such as comorbidities, medications or personal preferences, even though these are crucial for shared decision-making. Important aspects of peri-operative care, particularly general anaesthetic risks, were not consistently addressed. Finally, AI systems cannot perceive or respond to non-verbal cues such as anxiety, confusion or emotional distress, limiting their capacity to support the psychological needs of patients [20, 29].

## Conclusions

ChatGPT’s responses to common patient questions about distal biceps tendon rupture and repair were judged to be of fair quality but required a relatively advanced reading level, with an FKGL of 11.8. The complete absence of authorship details, references and conflict-of-interest disclosures undermines transparency and makes it difficult to verify the information presented. While ChatGPT often encouraged users to consult a surgeon, it should be considered a supplementary source of general information rather than a replacement for clinician-led counselling. There is a continuing need for high-quality, evidence-based patient education materials that are transparently sourced and written in accessible language for individuals undergoing distal biceps tendon repair.

## Data Availability

The data generated and analysed during the current study are included within the manuscript and its Appendix. These datacomprise the 25 patient questions submitted to ChatGPT, the unedited ChatGPT responses, and the corresponding quality andreadability assessments. No patient-level, identifi able, or confi dential data were used. Further details are available from thecorresponding author upon reasonable request.

## Appendices

**Table:**

Question	Answer
How does an injury to the distal biceps occur?	An injury to the distal biceps typically occurs when the tendon attaching the muscle to the forearm is torn or ruptured. This often happens during sudden, forceful elbow extension or lifting heavy objects
How will I know if I’ve injured my biceps tendon?	Symptoms of a distal biceps tendon injury include sudden, severe pain in the elbow or forearm, weakness in the affected arm, bruising or swelling near the elbow, and a bulge in the upper arm due to the retracted muscle
Do I need surgery to repair the distal biceps tendon?	Surgery is often recommended to repair a torn distal biceps tendon, especially for younger, active individuals or those who require full arm strength and function
Who should get distal biceps repair surgery?	Individuals with a complete distal biceps tendon tear or rupture, particularly those who want to regain full arm strength and function, are often candidates for surgery
Can my distal biceps rupture be managed without surgery?	While some individuals may opt for non-surgical treatment such as rest, ice, physical therapy, and lifestyle modifications, surgery is generally recommended for optimal recovery and function
Will I need to stay overnight after surgery?	In most cases, distal biceps repair surgery is performed outpatient, meaning you can go home the same day
How long does the surgery take?	The surgery typically takes 1 to 2 h to complete, depending on the severity of the injury and the chosen surgical technique
Are there risks involved with having surgery?	Like any surgery, distal biceps repair involves risks, including infection, nerve damage, stiffness, and tendon re-rupture
What is done during surgery?	During surgery, the torn or ruptured distal biceps tendon is reattached to the bone using various techniques, such as suture anchors or tendon grafts
Are there different types of distal biceps tendon repair surgery?	Yes, there are different surgical techniques for repairing a torn distal biceps tendon, including single-incision and double-incision approaches
Which type of surgical repair is better?	The choice of surgical repair depends on factors such as the extent of the injury, the patient’s anatomy, and the surgeon’s preference. Both single and double-incision techniques can be effective
How long is the recovery after distal biceps repair?	Recovery time varies, but most patients can expect to return to light activities within a few weeks and resume full activities within 4 to 6 months after surgery
Will I need a brace after surgery?	A brace or splint may be prescribed initially to protect the repaired tendon, but it is typically worn for a few weeks and gradually discontinued as the arm heals
Will physical therapy or occupational therapy be needed after distal biceps repair?	Yes, physical therapy is usually recommended to help restore strength, flexibility, and range of motion in the affected arm
What medications will be prescribed after surgery?	Pain medications and anti-inflammatory drugs may be prescribed to manage post-operative pain and swelling
How much pain am I going to have after surgery?	Post-operative pain varies from person to person but is usually managed effectively with medications and proper post-operative care
When can I begin driving after distal biceps repair?	You should avoid driving until you have regained sufficient strength and range of motion in your arm, typically within 4 to 6 weeks after surgery
When can I return to work after distal biceps repair?	The timing of returning to work depends on the type of work you do. Most people can return to desk jobs within a few weeks but may need more time for physically demanding occupations
When can I return to sports after distal biceps repair?	You can usually return to sports and other strenuous activities within 4 to 6 months after surgery, following clearance from your surgeon and completion of physical therapy
Will distal biceps repair allow my elbow full function once recovered?	With proper rehabilitation, most patients can regain near-normal strength and function in the affected arm following distal biceps repair surgery
What do I do with the dressings after surgery?	Follow your surgeon’s instructions for changing dressings and keeping the surgical site clean. If you have any concerns about the dressing, contact your healthcare provider
Will I be able to return to the same activities after surgery that I was doing before surgery?	In many cases, yes. However, it’s essential to follow your surgeon’s recommendations for post-operative care and rehabilitation to optimise your recovery and minimise the risk of re-injury
Can the injury happen again?	While distal biceps repair surgery can significantly reduce the risk of re-injury, it’s essential to continue to practice proper lifting techniques and avoid overloading the affected arm to minimise the risk of recurrence
Can this injury occur in my opposite arm?	Yes, although less common, a distal biceps tendon tear can occur in the opposite arm, especially if you have predisposing factors such as repetitive strain or overuse
How successful is revision surgery?	Revision surgery for a failed distal biceps repair can be successful in many cases. Still, the outcome depends on various factors, such as the reason for the failure, the condition of the surrounding tissues, and the patient’s overall health. It’s essential to discuss the risks and benefits with your surgeon
